# Variation in the ratio of compounds in a plant volatile blend during transmission by wind

**DOI:** 10.1038/s41598-022-09450-z

**Published:** 2022-04-13

**Authors:** Xiaoming Cai, Yuhang Guo, Lei Bian, Zongxiu Luo, Zhaoqun Li, Chunli Xiu, Nanxia Fu, Zongmao Chen

**Affiliations:** Key Laboratory of Tea Biology and Resource Utilization, Ministry of Agriculture, Tea Research Institute, Chinese Academy of Agricultural Science, Hangzhou, 310008 China

**Keywords:** Chemical biology, Ecology

## Abstract

For plant volatiles to mediate interactions in tritrophic systems, they must convey accurate and reliable information to insects. However, it is unknown whether the ratio of compounds in plant volatile blends remains stable during wind transmission. In this study, volatiles released from an odor source were collected at different points in a wind tunnel and analyzed. The variation in the amounts of volatiles collected at different points formed a rough cone shape. The amounts of volatiles collected tended to decrease with increasing distance from the odor source. Principal component analyses showed that the volatile profiles were dissimilar among different collection points. The profiles of volatiles collected nearest the odor source were the most similar to the released odor. Higher wind speed resulted in a clearer spatial distribution of volatile compounds. Thus, variations in the ratios of compounds in odor plumes exist even during transport over short distances.

## Introduction

Volatile compounds emitted from plants are a chemical language representing plant signals, and they play an important role in mediating interactions in tritrophic systems^1,2^. Herbivores use plant volatiles to find food plants or sites for egg deposition^3,4^, pollinators use plant volatiles to locate flowers^5,6^, and carnivorous or parasitic insects also use plant volatiles to find herbivorous prey and hosts^7,8^. Plant volatiles exhibit immense diversity and variability. Approximately 1700 different plant volatile compounds have been identified and are produced by plants in more than 90 families^9^. The quantitative and qualitative composition of plant odors differs among plant species, with changes in the physiological state of the plant, and with different biotic stresses and abiotic stresses^10,11^. Thus, plant volatiles, as a “chemical language”, can convey information from the plant to pollinators, predators, parasitic wasps, and herbivorous insects^1,2^. Although plant volatiles can contain hundreds of components, only a small subset of volatile compounds (10 compounds or fewer) is used by insects for host location, and the ratios of these compounds play an important role^4,12–14^.

**Figure 1:**
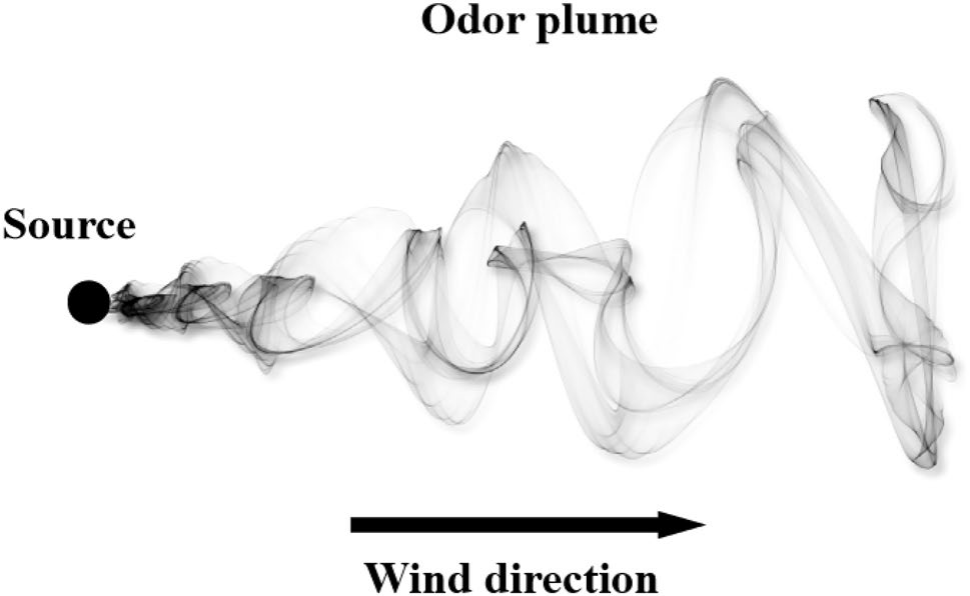
Simplified diagram of an odour plume. The odor plume from the source moves downwind.

After volatiles are emitted from plants, they are transmitted through the air before being perceived by insects. Volatiles spread away from their source through molecular diffusion and ambient motion^15,16^. Molecular diffusion is the net movement of molecules from a region of high concentration to a region of low concentration as a result of their random motion. This diffusion method transports odorants on small spatial scales over long time periods (ca. 80 min for an odorant molecule to travel 1 m)^17^. Ambient motion can transport odorant molecules > 10^3^ times quicker than molecular diffusion over equivalent distances and is the principal physical process that controls odor transport at distances > 1 cm^17^. Wind is the strongest dispersion factor for odors in natural landscapes and is turbulent. It carries plant odors as a plume that contains intermittent odor filaments (pockets of high odor concentrations) interspersed with patches of clean air (Fig. 1)^18–21^, much like the patterns observed in plumes of smoke.

Although plume-tracking insects use the wind direction as the primary directional cue to steer their movements toward the odor source^22–24^, a nearly universal strategy for odor-source location of insects is upwind locomotion modulated by moment-to-moment encounters with individual odor filaments, with each encounter resulting in an upwind surge^25–36^. Therefore, it is important to a recipient insect that the information carried by odor filaments should be relatively fixed and stable after turbulent transport in air. In other words, the qualitative and quantitative composition of the volatile compounds conveyed in the odor filaments should be conserved over long distances^1,35,37^, although the concentration of the odor within the filaments may decrease^21,38^. However, little is known about changes in the qualitative and quantitative composition of plant odors as they are transmitted by wind. This is mainly because it is difficult to obtain robust and reliable measurements of the instantaneous changes in plant odor composition that occur in plant odor filaments^39^. The averaged odor concentration in some time at different locations downwind of the source could also be used to evaluate the stability of the information carried by the plant odor plume^20^. In this study, the slow-releasing volatiles in a wind tunnel were collected at different points over a period of time. These samples were analyzed to evaluate the decrease in the concentration of the volatiles over a distance from a point source and the change in the qualitative and quantitative composition of the volatiles at different locations downwind of the source under different windspeeds.

## Results

### Variation in amounts of collected volatiles among different points

**Figure 2:**
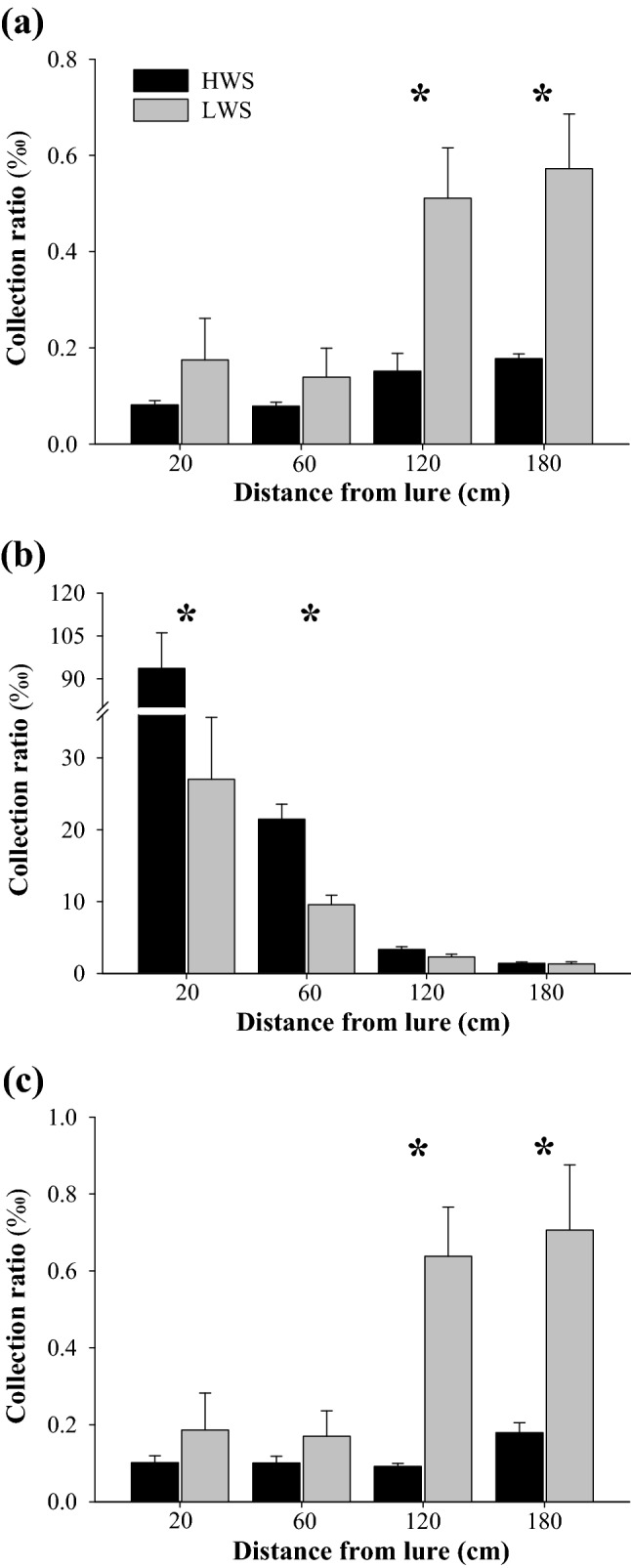
Collection ratios of total amounts of collected volatiles at different points in the wind tunnel under low and high wind speed. Collection ratio = (amount collected at a point/amount released from the source) × ‰. (**a**)–(**c**) indicate, respectively, left, middle and right, and are the horizontal position of the collection points compared with odor source. Value on abscissa indicates vertical position of the collection points from source odor in the downwind direction. HWS, high wind speed; LWS, low wind speed. Data are mean + SE (*n* = 4). Asterisks denote significant difference in collection ratio at a particular point between low and high wind speed (two-sample *t*-test for means: *P* < 0.05).

**Figure 3:**
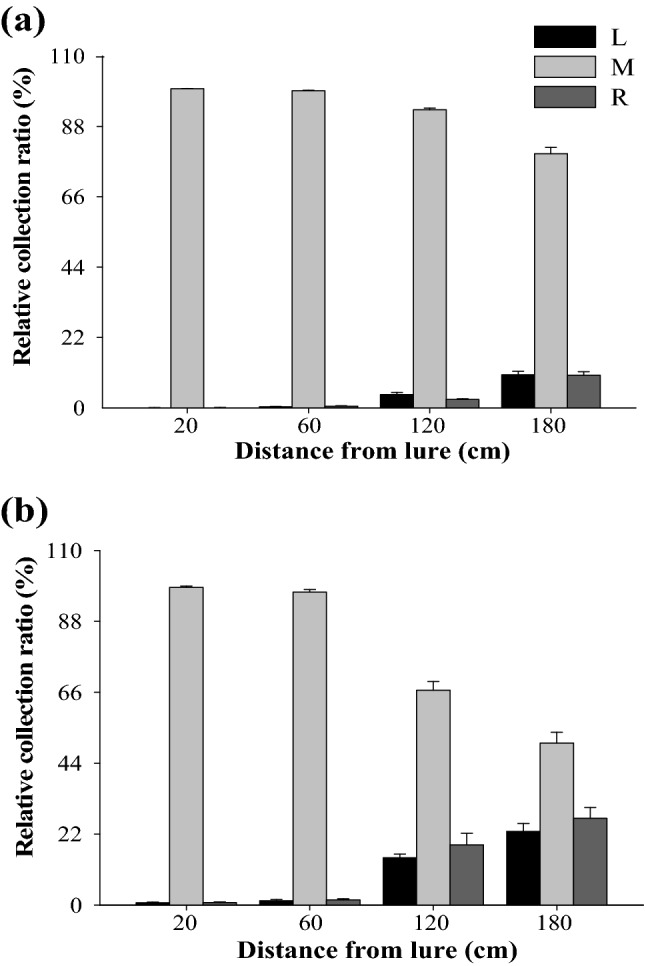
Relative collection ratio of total amount of collected volatiles at different points in the wind tunnel under high (**a**) and low (**b**) wind speed. Relative collection ratio = (collection ratio at a point/total collection ratio of the three points at the same horizontal distance from the odor source) × %. L, M, and R indicate, respectively, left, middle and right, and these are the horizontal position of the collection points compared with the odor source. Value on abscissa indicates vertical position of the collection points from odor source in the downwind direction. Data are mean + SE (*n* = 4).

In this study, the slow-releasing volatiles in a wind tunnel were collected for some time at twelve different points. The amount of volatiles collected at the middle four points exponentially decreased with increasing distance from the odor source (Fig. 2). In contrast, the collection ratios at right and left points increased with increasing distance from the odor source (Fig. 2). The collection ratios at M-20 (middle-20 cm) and M-60 (middle-60 cm) were, respectively, 87.1‰ and 27.3‰ under high wind speed, and were significantly higher than their corresponding values under low wind speed (*P* < 0.01, Fig. 2). The collection ratios at L-120 (left-120 cm), L-180 (left-180 cm), R-120 (right-120 cm), and R-180 (right-180 cm) were significantly lower under high wind speed than under low wind speed (*P* < 0.05, Fig. 2). The relative collection ratios at M-20, M-60, M-120 (middle-120 cm) and M-180 (middle-180 cm) were, respectively, 99.8%, 99.3%, 93.4% and 78.2% under high wind speed, and were all significantly higher than their corresponding values under low wind speed (*P* < 0.05, Fig. 3).

### Differences in profiles between released and collected volatiles

**Figure 4:**
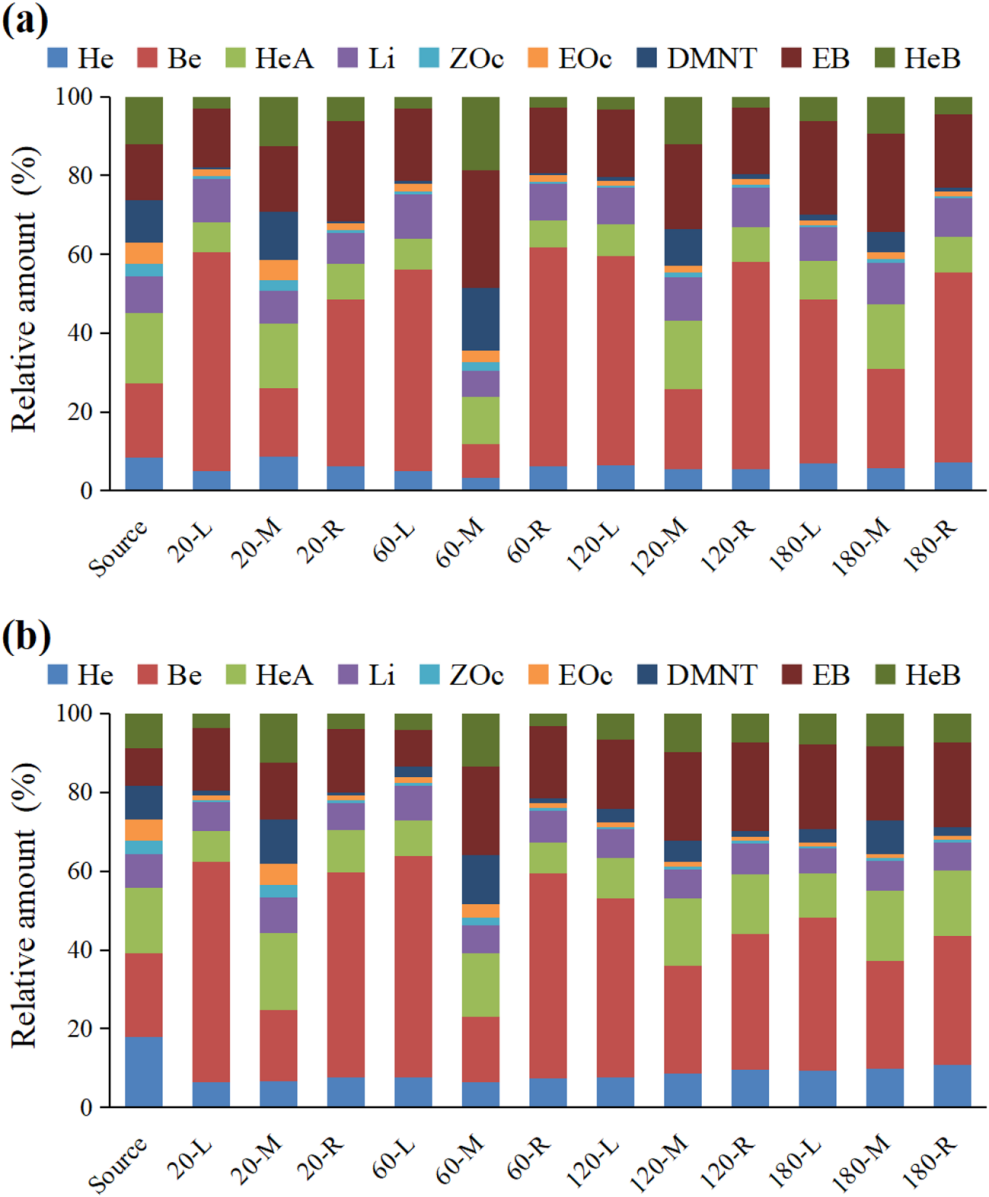
Profiles of volatiles released from the odor source and those collected at 12 points in the wind tunnel under high (**a**) and low (**b**) wind speed. L, M, and R indicate, respectively, left, middle and right, and these are the horizontal position of the collection points compared with the odor source. 20, 60, 120, and 180 indicate, respectively, 20 cm, 60 cm, 120 cm, and 180 cm vertical distance of collection point from odor source in the downwind direction. Source indicates the odor source. Nine compounds were as follows: (*Z*)-3-hexenol (He), (*Z*)-3-hexenyl acetate (HeA), (*Z*)-3-hexenyl butyrate (HeB), benzaldehyde (Be), ethyl benzoate (EB), limonene (Li), (*E*)-β-ocimene (EOc), (*Z*)-β-ocimene (ZOc), and (*E*)-4,8-dimethyl-1,3,7-nonatriene (DMNT). Data are the percentages of compounds out of total collected or released volatiles, and are mean for four replicates.

**Figure 5:**
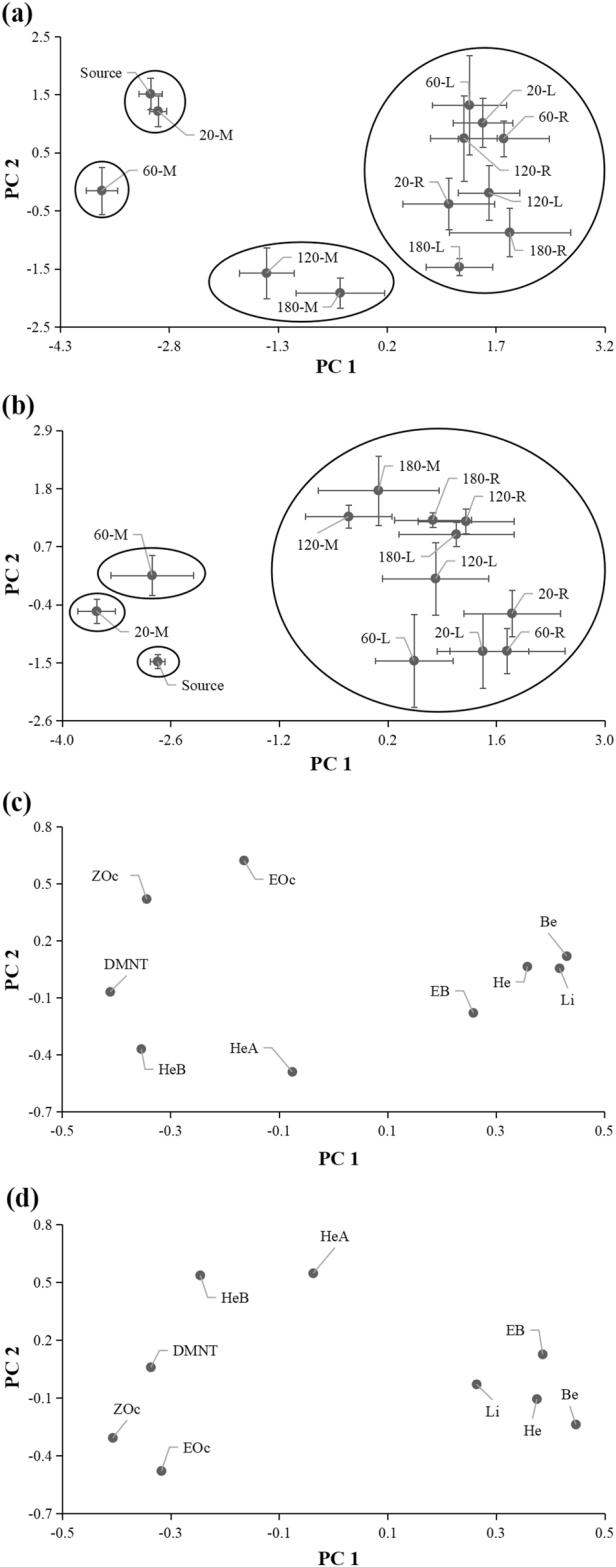
Principal component analysis (PCA) of profiles of volatiles released from the odor source and those collected at 12 points in the wind tunnel under high (**a** score plot; **c** loading plot) and low (**b** score plot; **d** loading plot) wind speed. L, M, and R indicate, respectively, left, middle and right, and these are the horizontal position of the collection points compared with the odor source. 20, 60, 120, and 180 indicate, respectively, 20 cm, 60 cm, 120 cm, and 180 cm vertical distance of collection point from odor source in the downwind direction. Source indicates the odor source. Nine compounds were as follows: (*Z*)-3-hexenol (He), (*Z*)-3-hexenyl acetate (HeA), (*Z*)-3-hexenyl butyrate (HeB), benzaldehyde (Be), ethyl benzoate (EB), limonene (Li), (*E*)-β-ocimene (EOc), (*Z*)-β-ocimene (ZOc), and (*E*)-4,8-dimethyl-1,3,7-nonatriene (DMNT). Score plots from PCA are based on percentages of compounds out of total collected or released volatiles. Data points in the score plot are mean ± SE of principal component scores for four replicates.

Although all nine compounds were collected at the 12 collection points, the profiles of the volatiles at the odor source and at the 12 collection points were different (Fig. 4). The results of the PCA showed that the volatile profiles were divided into four groups under high wind speed: (1) odor source and M-20; (2) M-60; (3) M-120 and M-180; and (4) the remaining eight collection points (Fig. 5a). There was a short distance between Group 1 and Group 2. The first component (PC 1) accounted for 53.4% of the total variation, and distinguished the four groups, while PC 2 accounted for an additional 21.9% of the variation, and distinguished group 1, group 2, and group 3. An ANOVA of the PC 1 and PC 2 scores revealed significant differences in the volatile profiles (PC 1: *F*_horizontal_ = 124.99, *P*_horizontal_ < 0.0001; *F*_vertical_ = 5.45, *P*_vertical_ = 0.0016; *F*_horizontal*vertical_ = 5.73, *P*_horizontal*vertical_ = 0.0003. PC 2: *F*_horizontal_ = 3.47, *P*_horizontal_ = 0.0417; *F*_vertical_ = 14.97, *P*_vertical_ < 0.0001; *F*_horizontal*vertical_ = 3.69, *P*_horizontal*vertical_ = 0.0058). In loading plot, Group 1 was characterized by (*E*)-β-ocimene and (*Z*)-β-ocimene; Group 2 was characterized by DMNT; Group 3 was characterized by (*Z*)-3-hexenyl acetate and (*Z*)-3-hexenyl butyrate; and Group 4 was characterized by (*Z*)-3-hexenol, benzaldehyde, limonene, and ethyl benzoate (Fig. 5c).

Under low wind speed, the volatile profiles were separated into four groups: (1) odor source; (2) M-20; (3) M-60; and (4) the remaining 10 collection points (Fig. 5b). Group 1 was close to group 2. The first component accounted for 47.4% of the total variation, and distinguished between Group 1 and Group 3, and between Group 2 and Group 3. The PC 2 accounted for an additional 22.1% of the variation, and distinguished group 1 and group 2. An ANOVA of the PC 1 and PC 2 scores revealed significant differences (PC 1: *F*_horizontal_ = 83.94, *P*_horizontal_ < 0.0001; *F*_vertical_ = 5.29, *P*_vertical_ = 0.0019; *F*_horizontal*vertical_ = 11.90, *P*_horizontal*vertical_ < 0.0001. PC 2: *F*_horizontal_ = 6.78, *P*_horizontal_ = 0.0032; *F*_vertical_ = 20.96, *P*_vertical_ < 0.0001). In the loading plot, the characteristic compounds of each group under low wind speed were similar to those under high wind speed (Fig. 5d).

### Differences in the spatial distribution of volatile compounds

**Figure 6:**
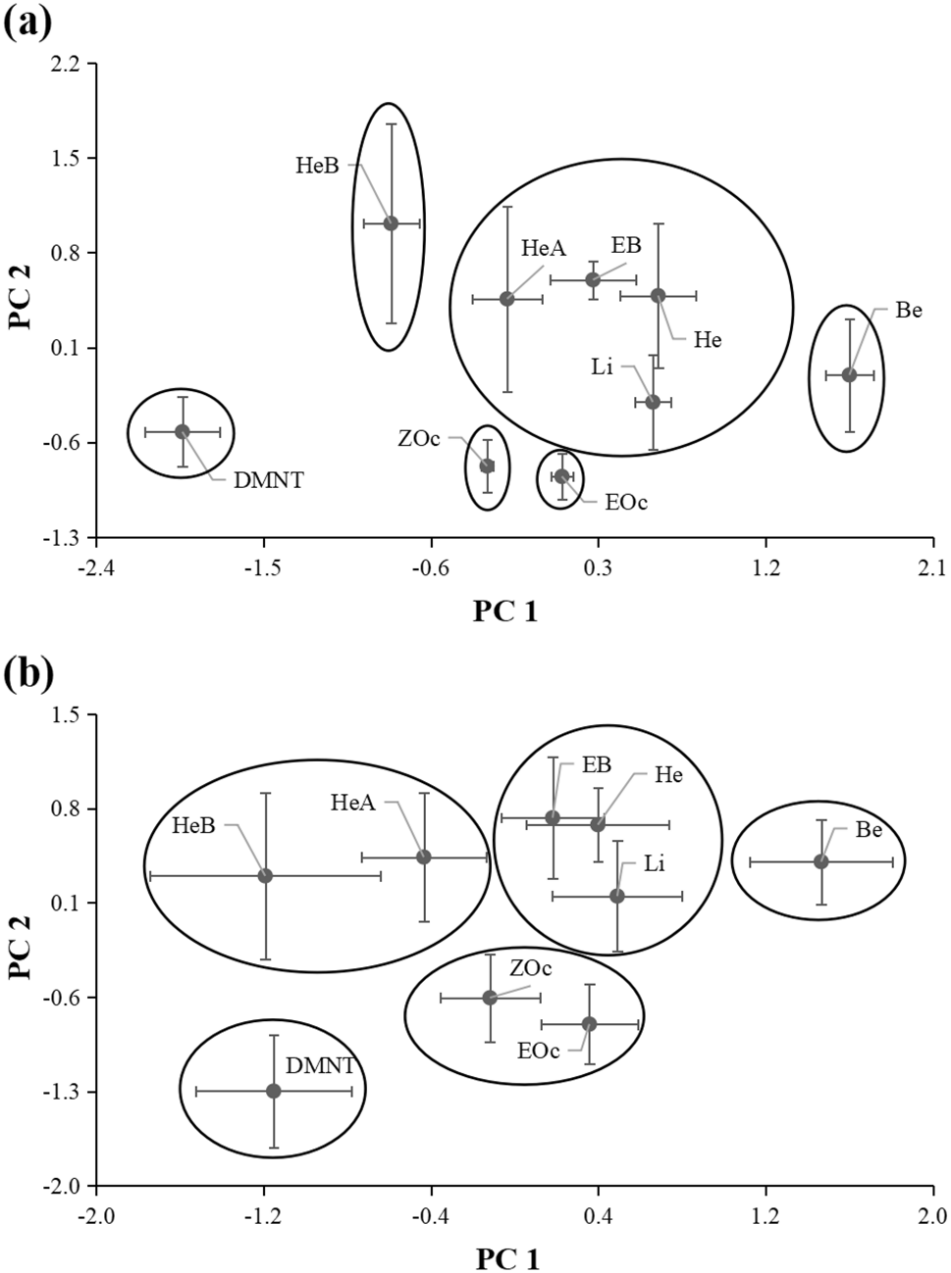
Principal component analysis (PCA) of spatial distribution of slow-released volatile compounds at 12 collection points in the wind tunnel under high (**a**) and low (**b**) wind speed. Score plots from PCA were based on collection ratios of the nine compounds. Collection ratio = (amount collected at a point/amount released from source) × ‰. Nine compounds were as follows: (*Z*)-3-hexenol (He), (*Z*)-3-hexenyl acetate (HeA), (*Z*)-3-hexenyl butyrate (HeB), benzaldehyde (Be), ethyl benzoate (EB), limonene (Li), (*E*)-β-ocimene (EOc), (*Z*)-β-ocimene (ZOc), and (*E*)-4,8-dimethyl-1,3,7-nonatriene (DMNT). Data points in the score plot are mean ± SE of principal component scores for four replicates.

The nine volatile compounds showed different spatial distributions in the wind tunnel. The results of the PCA showed that, under the high wind speed, the nine compounds were separated into six groups on the basis of their collection ratios: (1) DMNT; (2) benzaldehyde; (3) (*E*)-β-ocimene; (4) (*Z*)-β-ocimene; (5) (*Z*)-3-hexenyl butyrate; and (6) the remaining four compounds (Fig. 6a). The first component accounted for 58.2% of the total variation, and distinguished group 1, group 2, group 3, group 4, and group 5. The second component accounted for an additional 16.0% of the variation, and distinguished group 3 and group 4 from group 5 and group 6. An ANOVA of the PC 1 and PC 2 scores revealed significant differences among the nine compounds (PC 1: *F* = 40.75, *P* < 0.0001; PC 2: *F* = 5.31, *P* = 0.0007). The nine compounds were separated into five groups under low wind speed: (1) DMNT; (2) benzaldehyde; (3) (*E*)-β-ocimene and (*Z*)-β-ocimene; (4) (*Z*)-3-hexenyl butyrate and (*Z*)-3-hexenyl acetate; and (5) the remaining three compounds (Fig. 6b). PC 1 and PC 2 accounted for 41.6% and 23.8% of the total variation, respectively. The first component mainly distinguished group 1, group 2, and group 3; while PC 2 mainly distinguished group 2, group 4, and group 5 from group 1. An ANOVA of the PC 1 and PC 2 scores revealed significant differences among the nine compounds (PC 1: *F* = 15.95, *P* < 0.0001; PC 2: *F* = 14.96, *P* < 0.0001).

## Discussion

Plant odor-mediated insect navigation is vital for interactions in tritrophic systems^1,2^. It is important to a recipient insect that the information carried by the plant odor should be relatively fixed and stable. However, little is known about changes in the qualitative and quantitative composition of plant odors as they are transmitted by wind. In this study, the slow-releasing volatiles in a wind tunnel were collected at different fixed points and analyzed to evaluate the stability of the information carried by plant odor. The collected amounts of volatiles at different points formed a rough cone shape, and tended to decrease with increasing distance from the odor source. The volatile profiles were dissimilar among different collection points, and that a higher wind speed resulted in a clearer spatial distribution of volatile compounds. These results indicated that the ratios of compounds in plant odor could change during transmission by wind.

It has been reported that the an odor plume becomes wider and taller as it is carried away from the source, whereas the intermittency and intensity of odor filaments and the average concentration of the odor decreases with increasing distance from the source^20,40^. Thus, the odor gradients allow insects to track the odor plume to the location of an odor source^20,36^. In the present study, measurements of the amounts of volatiles collected in a two-dimensional space showed that the slow-released odor formed a rough cone shape in the direction of the blowing wind in the wind tunnel. Moreover, the amounts of volatiles exponentially decreased with increasing distance from the odor source. This spatial outline may indicate that insects are more likely to encounter the odor at sites more distant from the odor source because of the large diffusion area of volatiles. Then, they can follow the higher concentrations of volatiles to easily locate the odor source. In addition, the odor plume became a sharper cone with higher concentrations of volatiles under a higher wind speed. Thus, under a higher wind speed, the odor could be detected by insects further from the odor source.

We evaluated the stability of a volatile blend during wind transmission in a wind tunnel. The results of PCA analyses showed that the profiles of volatiles collected at different points were significantly dissimilar whether the wind speed was high or low, and that the spatial distribution of the nine volatile compounds was also significantly dissimilar (Figs. 5 and 6). These results indicate that the ratios of compounds in odor filaments could change during wind transmission. This change is unlikely to be caused by the atmospheric chemical reactions of some unstable compounds, such as limonene and ocimene, with O_3_, hydroxyl radicals and nitrate radicals in ambient air, because the travel time of the volatiles in the wind tunnel was very short, no more than 30 s^41,42^. This is an interesting result. After all, according to previous predictions^1,35^, steady and clean air flow in a wind tunnel should lead to relatively fixed volatile profiles among different collection points. Differences in the profiles of the collected volatiles may be related to the physical properties of volatile compounds, such as molecular mass, vapor density, vapor pressure, and etc.. These physical properties might affect the inertial forces, viscous forces, and buoyancy forces of volatile compounds in ambient motion by air^26,43^. This is analogous to the different movement speeds of objects with different shapes or weight in flowing water, where larger differences in relative distance among objects occur with faster water flow and at sites further from the starting point. More research is required to explore if, and how, the physical properties of different compounds affect the ratios of volatile compounds in odor plumes.

The variations in the ratios of compounds in odor plumes will be larger in the field than those in this study, because of turbulent air flow, mixtures of different odors, adsorption by substrates, and atmospheric chemical degradation^44,45^. For instance, the ratio of volatiles in the plume emanating from flowers of *Datura wrightii* was found to change with distance, as the background volatiles from neighboring vegetation became intermixed with *D*. *wrightii* volatiles^46^. But insects such as moths can fly over several hundreds of meters navigating upwind through pheromone plumes^47–49^. It may be acceptable to the plume-tracking insect that the compound ratios in odor filaments change within a certain range. However, it has been postulated that insects can deal with complex odors because of their remarkable capacity to processes the fine-scale spatial and temporal patterns of odorant concentrations, which includes active olfactory sampling behaviors and self-generated airflows^26,50,51^. Of course, it is also possible that variations in the qualitative and quantitative composition of plant odors could be perceived by foraging insects and used to determine the distance to an odor source. Further research should explore this topic in more detail.

The aerodynamic environment can dictate important ecological interactions and be a selective force for the evolution of olfactory systems, because it determines the chemical information available to an organism^16,36,52^. Further studies are required to clarify the factors affecting the availability of olfactory information, both in terms of the spatiotemporal variations of the odor concentration and the resolution of the insect’s olfactory system. The essential prerequisite for such research is highly sensitive analytical methods for the qualitative and quantitative analysis of instantaneous variations in odor filaments.

## Methods

### Standards of volatile compounds

The tested plant volatile compounds were selected on the basis of the previous studies, which identified the compounds that convey chemical information of host location to tea leafhopper (*Empoasca onukii*) and are attractive to tea leafhopper^53,54^. They are representatives of various classes of plant volatile compounds, including green leaf compounds [(*Z*)-3-hexenol, (*Z*)-3-hexenyl acetate and (*Z*)-3-hexenyl butyrate], phenylpropanoids/benzenoids (benzaldehyde and ethyl benzoate), and terpenoids [limonene, ocimene (mixture of isomers), and (*E*)-4,8-dimethyl-1,3,7-nonatriene (DMNT)]. These compounds were high-purity grade. DMNT was synthesized by Laviana Corporation (Taizhou, Jiangsu, China). Other volatile compounds and internal standard (IS, decanoic acid ethyl ester) were obtained from Sigma-Aldrich (China).

### Odor source

The tested compounds were mixed at equal volumes. Forty microliters of the mixture was loaded onto a rubber septum, which was then placed in a refrigerator at 4 °C. After the mixture was completely absorbed (about 10 h), the rubber septum was used for tests in the wind tunnel. Before each test, the rubber septum was placed in a fume cupboard at 20 ± 2 ℃ for 3 h.

### Wind tunnel

**Figure 7:**
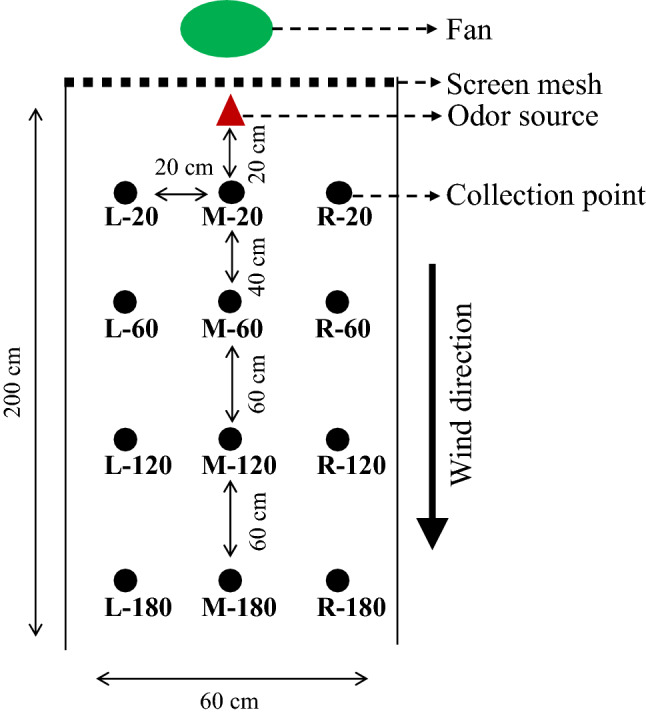
Schematic drawing of measurement of spatial distribution of slow-released volatiles in wind tunnel. L, M, and R indicate, respectively, left, middle and right; these are the horizontal position of the collection points compared with the odor source. 20, 60, 120, and 180 indicate, respectively, 20 cm, 60 cm, 120 cm and 180 cm vertical distance of collection point from odor source.

The wind tunnel had a polycarbonate flight section (length × width × height) of 200 cm × 60 cm × 60 cm (Fig. 7). Air was blown into the tunnel by a fan through an activated carbon filter and honeycomb-structured plastic, which were located inside a filter housing. The air exiting the tunnel was passed through a 100-mesh metal screen and a box filled with activated charcoal before being extracted from the room containing the wind tunnel via an exhaust system. Smooth airflow in the wind tunnel was confirmed by burning a mosquito coil at the site of odor source before each test. Hot-film anemometers (AR866, Dongguan Science & Technology Co. Ltd., Dongguan, China) were used to measure the wind speed at the five points near the exit of the wind tunnel. The wind tunnel was lit diffusely from above at about 200 lx. The room was kept at 20 ± 2 ℃, 70–80% relative humidity (R.H.). Before each test, the wind tunnel were cleaned with ethyl alcohol and maintained in a ventilated environment for 8 h.

### Volatile collection

The rubber septum was threaded onto a string with paper clips. The string was hung in the center of the flight section of the wind tunnel at the upwind end. There was a 20-cm distance between the rubber septum and the honeycomb-structured plastic, and a 30-cm distance between the rubber septum and the top of the wind tunnel. Samples were collected at 20 cm, 60 cm, 120 cm and 180 cm away from the odor source in the middle of the wind tunnel (four samples). Another eight samples were collected on the left and right sides of the middle four samples (at a distance of 20 cm from the middle sample). In total, samples at the 12 sites in the wind tunnel were simultaneously collected for 100 min (see Fig. 7). The air inlet of stainless steel adsorbent tubes (Markes, UK; packed with 200 mg of Tenax-TA, 60–80 mesh) was at about the same height as the odor source. Air was collected at a flow rate of 100 mL min^−1^ by using a microprocessor-controlled air sampling pump (Mini-pump Σ30; Shibata, Japan).

The trials included three treatments: low wind speed (0.09 m s^−1^), high wind speed (0.39 m s^−1^), and a blank control. In the low and high wind speed treatments, the odor sources were the same (rubber septa loaded with 40 µL volatile compound mixture). In the blank control, nothing was loaded on the rubber septum. Each treatment was replicated four times. Different treatments were tested on three consecutive days, and all of the wind tunnel tests were completed within 20 days. A new rubber septum was used for each test day, and all test were performed at 12:00 h.

To estimate the emission amount, the volatiles emitted from the same rubber septum as in the wind tunnel test were collected in a push/pull system at each time of the wind tunnel test. The rubber septum was maintained in a 30-mL glass holding chamber (2.1 cm i.d., 8 cm length). Charcoal-purified air entered the holding chamber at a rate of 100 ml min^−1^. After passing over the rubber septum, the air was pulled through the same stainless steel adsorbent tubes as those used in the wind tunnel test. Volatiles were collected at the 4th, 22nd, 40th, 58th, 76th, and 94th minute of the wind tunnel test, and each collection lasted for 2 min. Between collections, the flowing gas was retained in the chamber containing the rubber septum. The conditions of the collection room were the same as those used in the wind tunnel test.

### Volatile analysis

After collection, samples were analyzed immediately as described previously^39^. All samples were spiked with 5 ng IS, and were analyzed by coupled thermal desorption (TD; TD100, Marks, UK) and GC–MS (GCMS-QP2010, Shimadzu, Japan) with a DB-5 MS capillary column (60 m × 0.25 mm i.d., 0.25 µm film thickness; J&W Scientific, USA). The adsorbent tubes were heated at 275 °C for 5 min while the desorbed volatile compounds from the tube were focused into the cold trap at 4 °C with high-purity helium. Following sample transfer, the cold trap was rapidly heated to 290 °C. Then, the desorbed compounds were injected into the GC. The oven temperature of the GC was initially set to 45 °C for 2 min, then increased by 5 °C min^−1^ to 70 °C and held for 15 min, increased by 2 °C min^−1^ to 160 °C, and then increased by 30 °C min^−1^ to 260 °C and maintained for 10 min. Ionization was achieved via electron impact at 70 eV and 250 °C, and compounds were analyzed in the SIM mode. The calibration curve was established as described previously^39^ by plotting the abundance ratio of analyte to IS against the mass ratio of analyte to IS. The calibration curve was updated immediately before analyzing each batch of samples.

### Statistical analyses

The collection ratio and relative collection ratio were used to describe the dispersal behavior of slow-release volatiles in the wind tunnel. The formulae used to these ratios were as follows: Collection ratio = (amount collected at a point/amount released from the source) × ‰; Relative collection ratio = (collection ratio at a point/total collection ratio of the three points at the same horizontal distance from the odor source) × %. The amount released from the odor source was estimated as follows: 50 × average amounts detected at the 4th, 22nd, 40th, 58th, 76th, and 94th minute.

All statistical tests were carried out using SAS V8.2 (SAS Institute, Cary, NC, USA). Differences in the collection ratios or relative collection ratios at a collection point between high and low wind speed were determined using two-sample *t*-test for means. Prior to these analyses, the data were log_e_(*X* + 1)-transformed to satisfy the assumptions of normality and homogeneity. Principal component analysis (PCA) was used to compare the profiles of volatiles released at the odor source and those collected at different points, and to compare the collection ratios of nine compounds at different collection points^55,56^. For volatile profiles, the percentages of compounds in the collected or released volatiles were log_10_ (*X* + 0.00001)-transformed, mean centered, and represented as a covariance matrix before PCA. For collection ratios of compounds, the data were normalized, log_10_ (*X* + 0.00001)-transformed, mean centered, and represented as a covariance matrix before PCA. A mixed-model ANOVA of the factor scores for PCA was used to detect significant variations in the profiles of volatiles and the collection ratios of nine compounds. The ANOVA for the PCA based on the profiles included the horizontal position of the points (right, middle, and left), the vertical distance of the points from the source (0 cm, 20 cm, 60 cm, 120 cm, and 180 cm), and their interaction as fixed effects, and replicate as a random effect. The horizontal position and vertical distance of the source odor was, respectively, middle and 0 cm. For the PCA based on the collection ratios of nine compounds, compounds were considered as fixed effects and replicate as a random effect.
